# Small, Smaller, Smallest: Minimal Structural Requirements for a Fully Functional Box C/D Modification Guide RNA

**DOI:** 10.3390/biom9090457

**Published:** 2019-09-07

**Authors:** Svetlana Deryusheva, Joseph G. Gall

**Affiliations:** 1 Department of Embryology, Carnegie Institution for Science, Baltimore, MD 21218, USA; 2 Department of Biology, Johns Hopkins University, Baltimore, MD 21218, USA

**Keywords:** 2’-*O*-ribose methylation, box C/D snoRNA, guide RNA, *Xenopus*

## Abstract

Site-specific 2’-*O*-ribose methylation is an abundant post-transcriptional modification mediated by small non-coding nuclear RNAs known as box C/D modification guide RNAs. The minimal structural requirements for these guide RNAs to function in higher eukaryotes are still unclear. To address this question, we generated a series of mutant variants of *Drosophila* box C/D scaRNA:MeU2-C28 and tested their modification guide activities in the *Xenopus* oocyte system. Our data suggest that box C/D guide RNA function requires either a terminal or an internal consensus kink-turn structure. We identified the minimal functional box C/D guide RNA. It consists of a single-domain molecule with (i) a terminal stem with a consensus kink-turn domain, (ii) one box C and box D connected by a 14-nucleotide antisense element and (iii) a one-nucleotide spacer between the box C and the antisense element. In this single domain RNA, the sequence of the spacer is more important than its length. We suggest that the secondary structure of box C/D RNAs, essential for guide RNA function, is more complex than generally supposed. At the same time, the expression of functional extremely short single-domain box C/D RNAs is possible in higher eukaryotes.

## 1. Introduction

**Figure 1:**
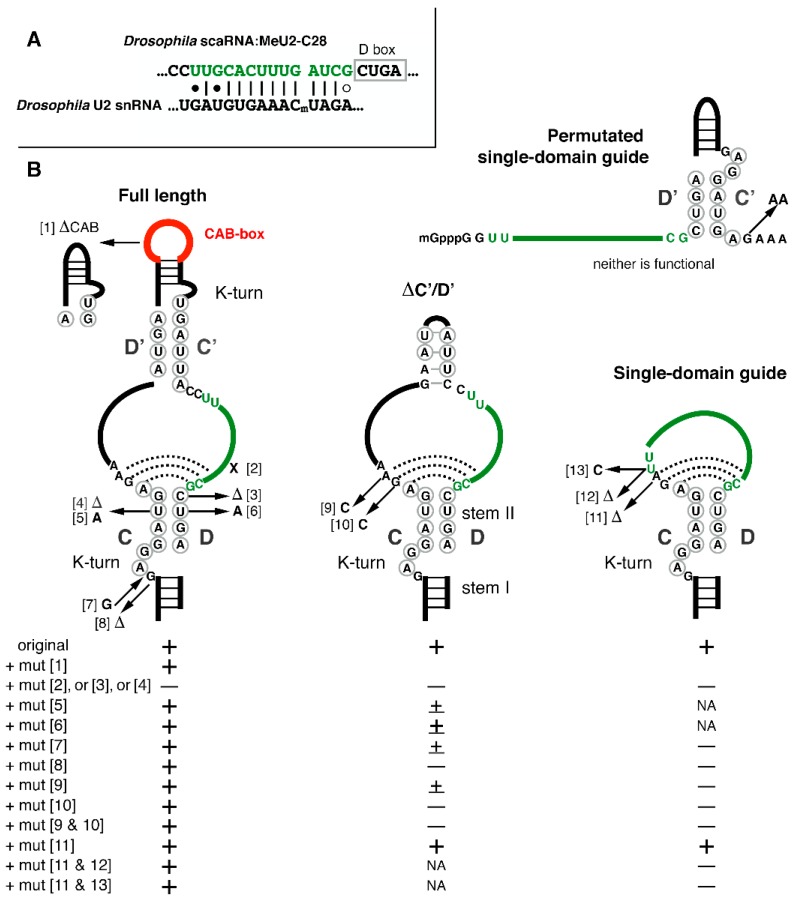
2’-*O*-methylation guide activity of *Drosophila* scaRNA:MeU2-C28 and mutants thereof, detected in injected *Xenopus* oocytes. (**A**) Postulated base-pairing of scaRNA:MeU2-C28 with U2 snRNA. (**B**) Schematic representation of tested MeU2-C28 variants. Experimentally verified guide activity or lack thereof are indicated with a plus or minus sign for each variant. Mutants that lacked the CAB box behaved like the corresponding full-length variants, thus confirming that the CAB box is dispensable for modification guide activity.

Small nucleolar RNAs (snoRNAs) are non-coding RNAs that may function as modification guide RNAs. Specifically, box C/D snoRNAs mediate site-specific post-transcriptional ribose methylation. The C and D boxes are two distinctive short motifs—‘RUGAUGA’ and ‘CUGA’, respectively. They localize close to the ends of a snoRNA molecule and participate in the formation of a specific terminal structure called the kink-turn, or K-turn. This structure consists of a canonical ‘stem I’ formed by 5’ and 3’ termini complementary to each other and a noncanonical ‘stem II’, which contains one U-U and two A-G pairs formed by the C and D boxes. Stems I and II are separated by an asymmetrical 3-nt bulge. Additional slightly diverged copies of the C and D boxes, the so-called C’ and D’ boxes, form an internal stem which is somewhat similar to the terminal one. Single-stranded sequences between the C and D’ boxes and the C’ and D boxes represent regions of complementarity with substrate RNAs targeted for modification. They are usually termed antisense elements (ASEs). The fifth nucleotide upstream of box D or D’ targets the position in the base-paired substrate RNA for modification. The typical structure of a box C/D snoRNA is depicted in Figure 1. The modification guide RNAs exist and function as RNP particles. Each box C/D snoRNP contains an RNA molecule and four core proteins: a 15.5K RNA binding protein (Snu13p in yeast, L7Ae in Archeae), Nop56p, Nop58p, and a methyltransferase called fibrillarin (yeast Nop1p). For additional general information on modification guide RNAs, see reviews by Bachellerie et al., Yu et al., and Watkins and Bohnsack [1,2,3].

Since the discovery of the snoRNA-guided mechanism for 2’-*O*-ribose methylation in eukaryotes [4,5,6], quite a few studies have focused on the structural features of box C/D snoRNAs that regulate snoRNA processing and accumulation or are required for snoRNP assembly, stability and function [7,8,9,10,11,12,13]. At least two previous studies attempted to determine the minimal core structure required for 2’-*O*-methylation activity [9,11]. Both studies used a yeast cell system to test snoRNA guide activities. Their findings were quite compelling, yet somewhat inconsistent.

One could argue that the RNA-guided modification machinery of higher eukaryotes, especially vertebrates, might have specific features different from those of yeast. Here we use a vertebrate cell system, *Xenopus* oocytes, to further explore box C/D RNA guide activity. This system has already been used successfully for verification of modification guide RNA activities [4,13,14,15]. We took advantage of scaRNA:MeU2-C28, a previously identified and tested *Drosophila* guide RNA for 2’-*O*-methylation of U2 snRNA at position 28 [13]. Because this position is not modified in vertebrates [13,16,17,18], there is no need for depletion of endogenous RNAs from the assay for guide activity. In our previous study, we showed that the CAB box—a short motif that targets RNA to a specific nuclear compartment, the Cajal body—is dispensable for modification guide RNA activity [13]. To further study functionally important structural domains, we systemically mutated *Drosophila* scaRNA:MeU2-C28 and tested the mutant RNAs for modification activity on U2 snRNA. In general, our data confirm the results of previous studies [7,11]. Moreover, in a single-domain box C/D guide RNA, we find that the primary sequence of the spacer between the ASE and the C box is more important than its length. In a symmetrical two-domain box C/D snoRNA, we postulate that the canonical internal C’/D’ K-turn structure can play a compensatory role in modification activity of the D box-associated ASE, when the terminal K-turn is structurally perturbed. These data suggest that structural interactions within eukaryotic box C/D snoRNPs are more complex than previously suspected.

## 2. Materials and Methods

### 2.1. Animals and Oocytes

*Xenopus laevis* females were anesthetized in 0.15% ethyl 3-aminobenzoate methane sulfonate (MS222; Sigma), and a piece of ovary was removed surgically. Oocytes were maintained in OR2 medium [19] for up to 5 days. All procedures were conducted in accordance with protocols approved by the Institutional Animal Care and Use Committee of the Department of Embryology, Carnegie Institution (Protocol approval #118).

### 2.2. In Vitro Transcription

DNA constructs were previously generated to make in vitro-transcribed *Drosophila* U2 snRNA and scaRNA:MeU2-C28, both wild type and mutants that lack the CAB-box [13]. Additional mutants of *Drosophila* scaRNA:MeU2-C28 were made by PCR-based mutagenesis and primer annealing-and-extension. Capped *Drosophila* sense-strand RNAs were transcribed in vitro from linearized plasmids using T3 RNA polymerase. DNA was removed by DNase I treatment followed by DNase I inactivation. RNAs were then purified on MicroSpin G25 columns (GE Healthcare). The integrity of the purified in vitro-transcribed RNAs was controlled by denaturing RNA gel electrophoresis. Small RNA and low range single-stranded RNA ladders (New England Biolabs) were used to estimate size of the in vitro-transcribed RNAs. Only RNA molecules that migrated on denaturing gels as one sharp band of the expected size with no sign of degradation and/or early termination were used for experiments. RNA concentration was measured with a NanoDrop spectrophotometer (Thermo Scientific, Wilmington, DE, USA).

### 2.3. In Vitro Modification Assays

We previously described in vitro modification assays using the *Xenopus* oocyte system [13]. Briefly, we used a Nanoject microinjection apparatus (Drummond Scientific, Broomall, PA, USA) to inject 9.2 nL (85 fmol) of an in vitro-transcribed guide RNA into the giant nucleus of *Xenopus* oocytes, followed by 18.4–23 nL (0.5 pmol) of *Drosophila* U2 snRNA into the cytoplasm. As a control, we injected U2 snRNA alone into the cytoplasm. Oocytes were incubated overnight in OR2 medium at room temperature and nuclei were then manually isolated in 5:1 KCl-NaCl isolation medium [20]. RNA was extracted using the RNAqueous-micro kit (Ambion) and the *Drosophila* U2 snRNA was tested for 2’-*O*-methylation induced by the injected guide RNAs.

In the second type of in vitro modification assay, 150 nuclei were manually isolated and collected in mineral oil. The nuclei were centrifuged to disrupt the nuclear envelope and release the nuclear contents. The resulting nuclear extract was treated with micrococcal nuclease (New England Biolabs) followed by addition of EGTA to inactivate the nuclease. In vitro-transcribed RNAs were added to the nuclease-treated nuclear extract. RNAs were either *Drosophila* U2 snRNA alone or U2 snRNA supplemented with a guide RNA. RNA was extracted after 6–12 h of incubation and analyzed for 2’-*O*-methylation.

### 2.4. 2’-O-methylation Mapping

2′-*O*-methylation was analyzed by a fluorescent primer extension-based method. We previously described a 6-FAM-labeled oligonucleotide specific for *Drosophila* U2 snRNA [13]. To detect 2’-*O*-methylation, we performed primer extension with a low concentration of dNTP (0.004 and 0.01 mM) along with control reactions at a high concentration of dNTPs (0.5 mM). AMV reverse transcriptase (New England Biolabs) was chosen to perform 2’-*O*-methylation analysis. Based on our previous experience [16], this enzyme is most suitable for reliable detection of U2-Cm28 in U2 snRNA modified in *Xenopus* oocytes. Fragments were separated on a capillary electrophoresis instrument (ABI Prism 3100 Genetic Analyzer; Applied Biosystems, Foster City, CA, USA) using parameters suggested by the manufacturer. To align fragments from different samples we added the Gene Scan-500 Liz Size Standard (Applied Biosystems) to each sample. We determined the exact positions of modified nucleotides with RNA sequencing products. In vitro-trans, cribed *Drosophila* U2 snRNA was used as a template. For chain termination, one dNTP was mixed with acyNTP (New England Biolabs) at a 1:3 (dNTP:acyNTP) ratio. Data were analyzed and visualized using GeneMapper software (Applied Biosystems).

Reverse transcription-based methods of 2’-*O*-methylation analysis are not truly quantitative and modification efficiency in *Xenopus* oocytes often depends on the frog used for assays. Therefore, we always injected experimental and control RNAs into oocytes from the same batch and performed enzymatic reactions on control and experimental RNAs simultaneously. For each experiment, we performed several replicates and ran serial dilutions of each sample on capillary columns.

### 2.5. Expression of Drosophila snoRNAs in HeLa Cells

**Figure 2:**
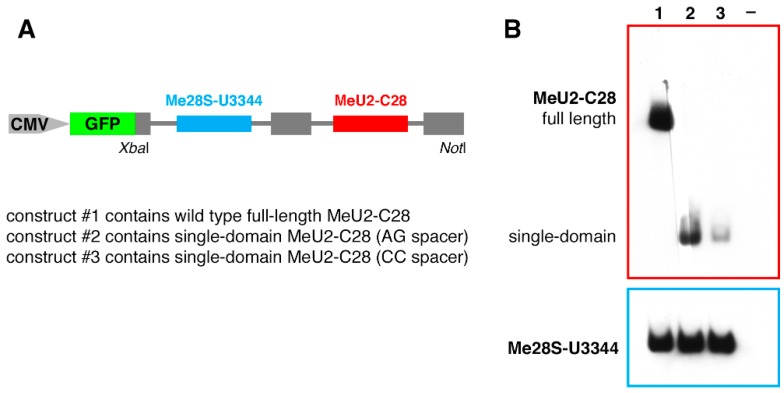
Expression of *Drosophila* snoRNA:Me28S-U3344 and scaRNA:MeU2-C28 (wild-type and single-domain mutant variants) in transfected HeLa cells. (**A**) Diagram of the snoRNA expression construct used for transfection. (**B**) Northern blot analysis of MeU2-C28 expression; Me28S-U3344 served as an internal control. Note fully processed guide RNA molecules in all samples. The expression level of the functional single-domain MeU2-C28 guide RNA is similar to that of full-length wild-type. A non-functional, single-domain mutant (extreme mutant with additional mutations 9 and 10) accumulated to a much lower level, yet was still normally processed.

A fragment of the *Drosophila Stt3A* gene was amplified from genomic DNA and cloned into the pCS2-GFP vector under a CMV promoter. This fragment contains introns 3 and 4, which encode snoRNA:Me28S-U3344a and scaRNA:MeU2-C28, respectively. In addition, two mutant variants were made in which scaRNA:MeU2-C28 was replaced with either an extremely short but still functional single-domain mutant or the same mutant with C in the 2-nt spacer between ASE and box C (shown schematically in Figure 1 and Figure 2A). Overlap extension PCR was used to generate mutated constructs.

These constructs were transfected into HeLa cells using the ViaFect transfection reagent (Promega). The transiently transfected cells were collected after 48 h cultivation and RNA was extracted using the TRIzol reagent. RNA was purified with the Direct-zol RNA MiniPrep Kit (Zymo Research, Irvine, CA, USA).

### 2.6. Northern Blot

Total RNA (3 μg per sample) was separated on 8% polyacrylamide–8M urea gels and transferred onto a nylon membrane (Zeta Probe, Bio-Rad). The expression of exogenous guide RNAs in HeLa cells was detected with digoxigenin-labeled probes. The probes were specific to *Drosophila* snoRNA:Me28S-U3344 and to the 3’ half of *Drosophila* scaRNA:MeU2-C28. Hybridization was performed at 40 °C for Me28S-U3344 detection and at 34 °C for MeU2-C28 detection in DIG Easy Hyb buffer (Roche). An anti-Dig antibody conjugated with alkaline phosphatase and the chemiluminescent substrate CDP-Star (Roche) were used to visualize the hybridized probes.

## 3. Results and Discussion

The major goal of our experiments was to determine the structural elements in box C/D guide RNAs that play critical roles in RNA-guided post-transcriptional 2’-*O*-methylation. For this purpose, we chose scaRNA:MeU2-C28, a *Drosophila* box C/D guide RNA. Earlier, we showed that this guide RNA is active in the *Xenopus* oocyte system [13]. Here, we started with the previously tested full-length wild type and ΔCAB mutants and generated over 40 additional variants. The variants are classified into four major groups shown in Figure 1B. Point mutations introduced into each of the major variants are depicted. In the interest of space, we do not include all intermediate variants generated and tested in this study.

**Figure 3:**
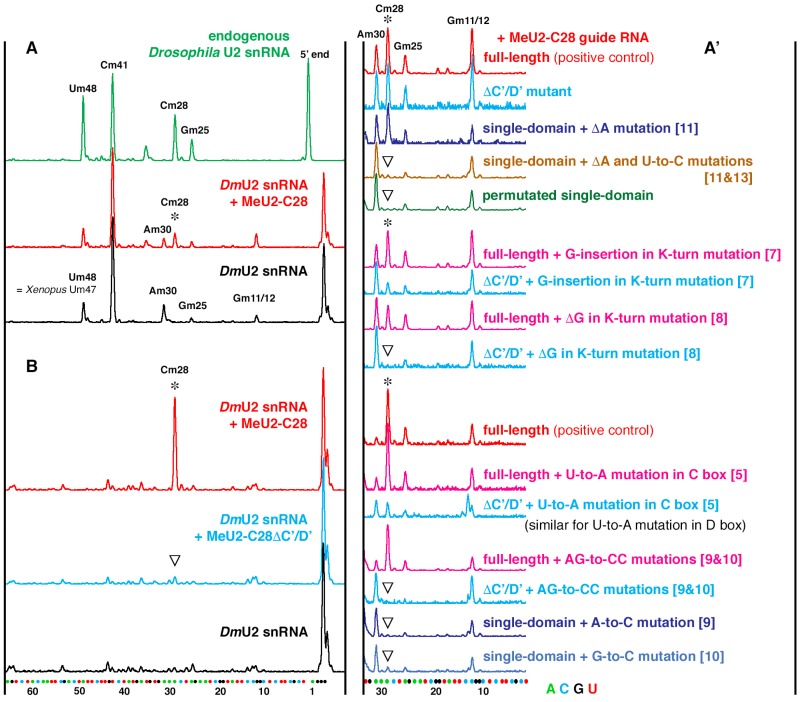
Mapping 2’-*O*-methylation of *Drosophila* U2 snRNA in in vitro modification assays. When dNTPs are used at low concentration, reverse transcriptase pauses at 2’-*O*-methylated positions and short fragments accumulate. The fluorescently labeled fragments appear as peaks above the baseline. The relative height of the peaks is significant only when oocytes from the same frog were used and modification assays were run at the same time (grouped traces). (**A**) 2’-*O*-methylation of endogenous *Drosophila* U2 snRNA (green trace) and in vitro-transcribed *Drosophila* U2 injected into *Xenopus* oocytes, either alone (black trace) or with in vitro-transcribed *Drosophila* MeU2-C28 guide RNA (red trace). Endogenous *Drosophila* U2 snRNA is modified at positions 48, 41, 28 and 25. When injected into *Xenopus* oocytes, *Drosophila* U2 snRNA becomes modified by the endogenous *Xenopus* modification machinery at position 30, specific to vertebrate U2 snRNA, but not at *Drosophila*-specific position 28. Vertebrate-specific 2’-*O*-methylation at positions 11 and 12 is also detected in *Drosophila* U2 snRNA injected into *Xenopus* oocytes (black trace). For more details on U2 snRNA modification patterns in vertebrates and *Drosophila*, see Supplementary Figure S1. Co-injection of MeU2-C28 induces 2’-*O*-methylation at position 28 (red trace, star). (**A’**) Zoom-in on the region of MeU2-C28-inducible modification. Traces show modification activity of representative mutants of MeU2-C28 guide RNA. Induced 2’-*O*-methylation at C28 is indicated with a star; unmodified C28 is indicated with an open arrowhead. (**B**) MeU2-C28 modification activity in RNA-depleted nuclear extract. Wild-type MeU2-C28 (top red trace) is functional in this assay, but the ΔC’/D’ mutant (blue trace) and other shorter mutants are not. As previously shown, the ΔCAB mutant is also functional in this assay [13].

As expected, the guide activity of *Drosophila* MeU2-C28 on U2 snRNA was completely eliminated by a point mutation in the ASE at the position targeting U2-C28 for modification (Figure 1, mutation 2), as well as by one-nucleotide deletions in the C (mutation 4) or D boxes (mutation 3). Surprisingly, mutations in the C and D boxes that involve replacement of the U-U base pair in the terminal stem II with canonical A-U and U-A base pairs (Figure 1, mutation 5 and 6, respectively) did not affect guide activity (Figure 1 and Figure 3A’, magenta traces). In nuclear extracts, these mutations were shown to reduce or abolish the binding of core snoRNP proteins Nop56, Nop58 and fibrillarin as well as chaperon proteins Tip48 and Tip49 to U14 box C/D snoRNA [10]. In our experiments, the same mutations reduced the guide activity in a variant of MeU2-C28 guide RNA, in which the C’/D’-box internal stem was mutated (ΔC’/D’ variant). The removal of the C’/D’ stem alone had little or no effect on the guide activity on U2 snRNA associated with D-box ASE (Figure 1 and Figure 3A’, light blue traces); the latter observation is in a good agreement with data reported by the Maxwell and Watkins laboratories [11]. How can we explain this unexpectedly well-preserved guide activity in full-length MeU2-C28 with U-to-A mutations in C and D boxes? One possibility is that in a cell-free in vitro system RNA–protein interaction is much less efficient than in living cells, which might affect proper RNP assembly. In fact, in our study, none of the mutant variants, except for the ΔCAB-box mutant, were functional in nuclear extracts (Figure 3B). We previously noticed limited guide RNA modification activities of a few other sno/scaRNAs in assays that used nuclear extracts from *Xenopus* oocytes instead of intact living cells [15].

Another possible explanation is that in U14, the C’/D’-box internal stem does not form a K-turn structure, whereas in MeU2-C28, both terminal and internal stems have typical K-turns (Figure 1). The asymmetrical structure of eukaryotic box C/D snoRNPs was revealed by site-specific cross-linking analysis [21]. However, two distinctive RNP complexes are formed with circularly permutated snoRNA U25 [22]. That is, when a snoRNA has the consensus terminal K-turn structure, the 15.5K protein binds only to the terminal domain. If the terminal K-turn is imperfect, an internal K-turn can take over in a snoRNP assembly process. In the *Xenopus* oocyte system, we definitely observe such a compensatory function, suggesting the existence of an interplay between two K-turns in the assembly of a functional snoRNP in vivo. To further confirm this hypothesis, we mutated the 3-nt bulge in the terminal K-turn structure. As predicted, a one-nucleotide insertion (Figure 1, mutation 7) or deletion within the bulge (mutation 8) did not change modification guide activity of the full-length MeU2-C28 (Figure 1 and Figure 3A’, magenta traces). However, the same one-nucleotide mutations dramatically reduced (mutation 8) or even completely abolished (mutation 7) the modification activity of the ΔC’/D’ guide RNA, which lacks C’ and D’ boxes (Figure 1 and Figure 3A’, light blue traces).

Thus, either a terminal or internal consensus K-turn is needed for eukaryotic box C/D snoRNA modification activity. The next question is: what is the minimal functional requirement for a 2’-*O*-methylation guide RNA in eukaryotes? We performed a serial deletion analysis of ΔC’/D’ MeU2-C28 and determined that the shortest functional guide RNA consists of a consensus terminal K-turn domain, a 14-nt ASE, and a one-nucleotide spacer between box C and the ASE (single-domain guide with mutation 11, Figure 1 and Figure 3A’, top dark blue trace). This is a considerably shorter functional guide RNA molecule than previously determined by the expression of artificial guide RNAs generated from human U14 box C/D snoRNA in a yeast cell system. In the previous study of a single-domain box C/D RNP function, shortening of the spacer from 8 to 4 nucleotides resulted in significant reduction of modification activity, and complete removal eliminated all activity [11]. However, we found that the sequence of the spacer between the ASE and box C is more important than its length; specifically, two nucleotides adjacent to the box C are the most critical. In full length MeU2-C28, these nucleotides (AG) could be mutated without effect on the guide RNA modification activity (Figure 1 and Figure 3A’, bottom magenta trace). However, in ΔC’/D’ and any shorter variants, the change of either A or G (or both) to C (mutations 9 and 10) completely eliminates guide activity (Figure 1 and Figure 3A’, bottom blue traces). This suggests that stem II structural interaction of the terminal K-turn domain might continue further into the postulated single-stranded regions between the C and D boxes. Accordingly, a U-to-C mutation in the ASE (mutation 13) of the extreme mutant MeU2-C28 makes this guide RNA non-functional (Figure 1 and Figure 3A’, brown trace). In this case, the binding affinity of the mutated ASE with the substrate U2 snRNA was not changed (Figure 1A). What might be affected is a potential base-pairing with the 3’ terminal region of the ASE. Thus, underappreciated minute differences in primary and secondary structure of a box C/D guide RNA can explain unexpected differences in modification guide activities. For instance, a C’/D’ unit inserted upstream of the box H/ACA snoRNA was found functional in one study [9] but non-functional in another [11]. We also did not detect modification activity when we tested the permutated box C’/D’ version of a single-domain MeU2-C28 guide RNA (Figure 1 and Figure 3A’, green trace). It is worth noting that endogenous snoRNAs with similar yet naturally functional domains have been identified in vertebrate species [23].

So far, our data confirm 2’-*O*-methylation activity of extremely short single-domain box C/D guide RNAs. The minimal functional RNA is basically a single box C and a single box D connected by a sequence of an ASE and flanked by short inverted sequences (Figure 1). Remarkably, the minimal guide RNA length is comparable to that of small silencing RNAs, like piRNAs, yet these tiny RNA molecules can form functional modification guide RNPs. One could argue that in vitro-transcribed mutated RNAs are stable when injected into *Xenopus* oocytes but would never be processed from a host gene intron and accumulate in the cell. To verify if single-domain snoRNAs can be processed naturally in cells, we generated several expression constructs (Figure 2A) with a fragment of *Drosophila* host gene for two guide RNAs: scaRNA:MeU2-C28 (experimental RNA) and snoRNA:Me28S-U3344 (internal control). We transfected HeLa cells with these constructs and analyzed the expression of *Drosophila* box C/D snoRNAs by northern blots. We found completely normal processing for both full length and extremely short single-domain MeU2-C28. An extremely short non-functional variant with a CC-spacer between the box C and the ASE (single-domain guide with mutations 9 and 10) accumulated at much lower levels, but otherwise was fully processed (Figure 2B). From our studies of snoRNA expression in the yeast cell system, we know that expression levels can vary dramatically without change of specific modification activities ([24], our unpublished observations). That is, decreased RNA accumulation alone cannot explain the loss of modification activity.

It was previously shown that all four core proteins of box C/D guide RNPs bind normally to a chimeric H/ACA snoRNA that contains only half of the box C/D domain [11]. Here, we show that fully-processed single-domain RNAs are stable and accumulate in cells, suggesting that overall RNP assembly is not significantly impaired. In both studies, some variants are functional as modification guide RNPs whereas other are not. Unfortunately, because in vitro assembly of *functional* eukaryotic box C/D snoRNPs is still challenging, detailed structural analysis of functional and non-functional variants is not feasible. Archaeal box C/D RNP complexes are accessible for the structural analysis. However, a symmetrical two-domain box C/D RNP is essential for function in Archaea [25,26,27,28]. Thus, for now it is not clear how minute changes in a single-domain box C/D RNA affect RNP structure and function in eukaryotes. Nevertheless, our data allow us to postulate that some very short box C/D snoRNA-like sequences found in eukaryotic genomes could represent functional, single-domain box C/D modification guide RNAs.

## 4. Conclusions

Our data strongly suggest that there are structural and functional interactions between the terminal and internal K-turn domains of box C/D guide RNAs. When one of the K-turns is mutated, the other K-turn protects the full-length guide RNA from loss of modification activity. At the same time, extremely short single-domain box C/D guide RNAs can also exist and function in the cell, although they are very sensitive to minute changes in their structure. What is the advantage, if any, of having such extremely short and vulnerable guide RNAs? How does a functional guide RNP particle assemble on these very short RNAs? Are there other functions of the short single-domain guide RNAs for which this size is crucial? These and other questions arising from our study still remain open.

## Supplementary Materials

The supplementary materials are available online https://www.mdpi.com/2218-273X/9/9/457/s1.
