# Supplementary material for: Small, Smaller, Smallest: Minimal Structural Requirements for a Fully Functional Box C/D Modification Guide RNA
Source: Biomolecules. 2019 Sep 7;9(9):457. doi: 10.3390/biom9090457 (PMC6770171; doi:10.3390/biom9090457)
Supplement: Supplementary file 1 [file biomolecules-09-00457-s001.pdf]

Supplementary Figure 1

U2 snRNA

```
                                                                40 42  45
             1                 25  28        35  38  41  44   48      55
Drosophila   AmU CGCUUCUCG G CCUUAUG GCUAAGmAUCmAA AGUGΨAGΨAΨCmΨGΨΨCUUmAUCAGCΨUAACAUC UGAUAGUUCCU  [1-73]
                         #                 #                                #     #          ## ###
Xenopus      AmUmCGCΨΨCUCGmGmCCΨUUUGmGCUAAGmAUC AAm-GUGΨAGΨAΨCmΨGΨΨCUUmAUCAGUΨUAAΨAUCmUGAUACGUCCC  [1-72]
                                                                                                 #
Human        AmUmCGCΨΨCUCGmGmCCΨUUUGmGCUAAGmAUC AAm-GUGΨAGΨAΨCmΨGΨΨCUUmAUCAGUΨUAAΨAΨCmUGAUACGUCCU  [1-72]
             1 2   6 7  11   15  19     25    30     34  37  40  43   47      54   58  61
                          12                                 39 41  44                 60
```

Alignment of the 5′ terminal sequences of U2 snRNA from *Drosophila, Xenopus* and human. The human sequence is identical to that of the mouse and other mammals. Mismatched nucleotides are indicated with a # sign. All known 2′-*O*-methylated positions (**Nm**) and pseudouridines (**Ψ**) are depicted. Modifications in *Drosophila*, human and mouse were previously mapped by Deryusheva and Gall 2009, 2017, Deryusheva et al. 2012; the modification pattern of *Xenopus* U2 snRNA was determined in this study. Note that **Cm28** is present in *Drosophila* but not in vertebrates, whereas positions **11, 12, 19 30** and **61** (equivalent to position 62 in *Drosophila*) are 2′-*O*-methylated in vertebrates but not in *Drosophila*.

We previously showed that termination of the reverse transcription reaction at 2′-*O*-methylated positions depends on the source of the enzyme (Deryusheva at al. 2012). AMV-RT from New England Biolabs, used in this study, is the best choice for testing modifications near C28. However, AMV-RT fails to stop at Gm19 and Cm61 in vertebrate U2. When we injected *in vitro* transcribed *Drosophila* U2 snRNA into *Xenopus* oocytes and looked for modifications, we found that Gm11/12, Gm25, Am30, Cm41 and Um48 were induced by the corresponding *Xenopus* guide RNAs; Gm19 and Cm62 (equivalent to position 61 in vertebrates) even if induced, would not produce stop signals and would not be detected in AMV-RT reactions.

References

Deryusheva, S.; Gall, J.G. Small Cajal Body-specific RNAs of Drosophila Function in the Absence of Cajal Bodies. *Mol. Biol. Cell* **2009**, *20*, 5250–5259.
Deryusheva, S.; Choleza, M.; Barbarossa, A.; Gall, J.G.; Bordonne, R. Post-transcriptional modification of spliceosomal RNAs is normal in SMN-deficient cells. *RNA* **2012**, *18*, 31–36.
Deryusheva, S.; Gall, J.G. Dual nature of pseudouridylation in U2 snRNA: Pus1p-dependent and Pus1p- independent activities in yeasts and higher eukaryotes. *RNA* **2017**, *23*, 1060–1067.
